# Lonely societies: low trust societies? Further explanations for national variations in loneliness among older Europeans

**DOI:** 10.1007/s10433-021-00649-z

**Published:** 2021-09-16

**Authors:** Gražina Rapolienė, Marja Aartsen

**Affiliations:** 1 Lithuanian Centre for Social Sciences, Vilnius, Lithuania; 2 Norwegian Social Research, Oslo Metropolitan University, Oslo, Norway

**Keywords:** Loneliness, Trust, Old age, Social engagement, Post-totalitarianism, Eastern and Central Europe

## Abstract

Cross-national studies in Europe reveal sharp regional differences in the prevalence of loneliness among older adults, with the highest prevalence of loneliness in Eastern European countries. In this study, we investigate an alternative explanation for differences in loneliness prevalence based on differences in trust. Many of the Eastern European countries were ruled by totalitarian regimes that undermined people’s trust in other people and in the system, potentially leading to higher loneliness prevalence. Data are derived from the sixth round of the European Social Survey conducted in 2012, based on 12,042 respondents, of which 4827 live in post-totalitarian countries and 7215 in other European countries and Israel. We estimate a path model with trust in people, trust in the system, and social engagement included as latent variables and one dichotomous outcome (lonely or not). We control for age, gender, health limitations, marital status, income adequacy, and education. The results reveal that loneliness is partly constructed by the social–cultural and historical–political characteristics of the countries in which people live. The higher prevalence of loneliness in the Eastern-European post-totalitarian countries can be linked to a low level of trust in other people through social disengagement. Considering the role of trust in the creation of individuals feelings of loneliness contributes to the understanding of country variations in loneliness and opens a new perspective in loneliness research and the development of policies aimed at reducing loneliness.

## Introduction

Loneliness is a negative experience that occurs when there is a discrepancy between desired and achieved levels of social contact (Perlman and Peplau 1982). Research has shown the significance of loneliness for health because of the harmful impact on mental and physical health (Cornwell and Waite 2009), weakening of cognitive functions and the increased risk of dementia in older age (Shankar et al. 2013), attempts at suicide (Waern et al. 2003), and increased mortality (Holt-Lunstad et al. 2015). However, the risk of being lonely is unequally distributed across countries, with the smallest share of lonely older people in Northern Europe (up to 6%) and the largest shares in Russia and Eastern Europe (10–34% in different age groups) (Yang and Victor 2011; Hansen and Slagsvold 2015). While loneliness is most prevalent among the oldest old, the rates of lonely *young* people in Eastern European countries are higher than the rates of lonely *old* people in Western European countries (Yang and Victor 2011). The increasing number and share of older people in contemporary societies and the unequal distribution of loneliness across countries make loneliness research among older adults a relevant scientific and public health topic. This study aims to shed more light on differences in loneliness prevalence between European countries.

Previous studies have pointed towards possible mechanisms behind cross-national differences in loneliness prevalence among older adults. Fokkema et al. (2012) suggest that the geographical divide in loneliness prevalence might be largely attributable to the demographic composition of countries—e.g. gender differences in life expectancy and variations in the share of widows. Others argue that loneliness is more common among the disadvantaged—i.e. those with lower socio-economic status, poorer health, and without partner (Hansen and Slagsvold 2015)—which may explain why Western European countries have lower loneliness prevalence than Eastern and Central European countries. Yet others suggest that country differences in loneliness prevalence could be attributed to cultural differences in relationship expectations in individualistic countries (often Northern European) versus collectivistic countries (often Southern European) (Jylhä and Jokela 1990; Dykstra 2009). Studies estimating the strength of the associations in multivariable models indicate that cultural differences in relationship expectations are associated with loneliness, but only partially (Lykes and Kemmelmeier 2014; Swader 2018).

The question this study seeks to answer is whether the higher prevalence of later life loneliness in Eastern and Central European countries can be (partly) attributed to the lower levels of trust in these countries. As part of the Eastern Bloc, Eastern and Central European countries were under the influence of the Soviet regime until 1991, when the Soviet Union collapsed and communist parties lost authority in Eastern and Central Europe. The Soviet regime can be dated back to the revolution of 1917, or even to 1914 and World War I, when it became possible to implement a Marxist programme for the reorganization of society (Hobsbawm 2000). To be more precise, due to terror and repressions of Stalin, the social reality in the Soviet Union was purely authoritarian from 1929 to 1953, and in Central Europe from 1948 to 1953. This totalitarian regime was maintained under Khrushchev (1954–1964) and Brezhnev (1964–1982), but without the terror that characterized Stalin’s regime (Norkus 2008). Despite decades having passed since the collapse of the Soviet Union, contemporary psychologists studying trauma conclude that the experience of living under a totalitarian regime is still insufficiently discussed in the public realm (Gailienė 2008). This lack of public discussion and the nature of the trauma may explain why even twenty years after the fall of the Soviet Union, in 2011, trust was not yet restored (Sapsford et al. 2015). Older people are the most impacted by the Soviet regime, as they spent most of their lives under these circumstances.

Fundamental shattering of (basic) trust—in oneself, in others, and in the world—can be caused by violence (Endress and Pabst 2013). Violence here is understood in a broad sense as negation of sociality (including forced labour, military drills, suppression of free expression, structures of political and juridical discrimination, ethnical or cultural stigmatization) and traumatic experiences. The totalitarian Soviet regime was built on violence and fear and characterized by deportations, political persecutions, and the total surveillance of private lives (Anušauskas et al. 2005; Courtois et al. 2000), which drove out not only trust in the state, but also trust in other people. No sphere of social life was free from state control as the distinction between the public and the private sphere lost its meaning in Marxist societies (Norkus 2008). Even some thirty years later, post-totalitarian states are still characterized by low levels of trust in the public realm alongside higher levels of trust in the private networks (Sapsford et al. 2015; Schrader 2004).

Not many studies among older adults have explicitly investigated the association between trust and loneliness, but there are reasons to expect such a relation. Psychodynamic theories about loneliness (Fromm Reichmann 1959; Hojat 1987) point at the relevance of childhood experiences in the genesis of loneliness. People are born with a need for contact and tenderness, but if this longing for intimacy is not satisfied because of a lack of love, loneliness arises. Similarly, the interactionist perspective on loneliness entails that if an attachment figure is absent, loneliness arises (Weiss 1973). Based on a review of theories on loneliness, Ernst and Cacioppo further suggest that insecure attachment that started in childhood accumulates over the years as those infants fail to develop age-appropriate social skills that hinders attachment to other people (Ernst and Cacioppo 1999). The current generation older people in former totalitarian states were disproportionately exposed to violence in their childhood, which coincided with the peak of the violent totalitarian regimes, which disrupted emotional bonds. Therefore, higher loneliness prevalence may be expected in (former) totalitarian states.

We are aware of a few experimental studies on the relationship between trust and loneliness with college students (Rotenberg 1994) and younger people aged 5–21 years (Rotenberg et al. 2010). These psychosocial studies indicated that loneliness is inversely related to different types of trust (Rotenberg 1994). This association is mediated, in part, by social disengagement, i.e. a lack of integration in social networks and relationships (Rotenberg et al. 2010). Nyquist and others evaluated associations between trust and loneliness in older people from a sociological perspective. Based on Putnam’s definition of social capital, they identify trust as one of the indicators of social capital and conclude that low social capital, especially in terms of low trust, is a risk factor for loneliness (Nyquist et al. 2016). We therefore expect trust to be a key explanatory factor for the higher prevalence of loneliness in many of the Eastern European countries.

**Fig. 1:**
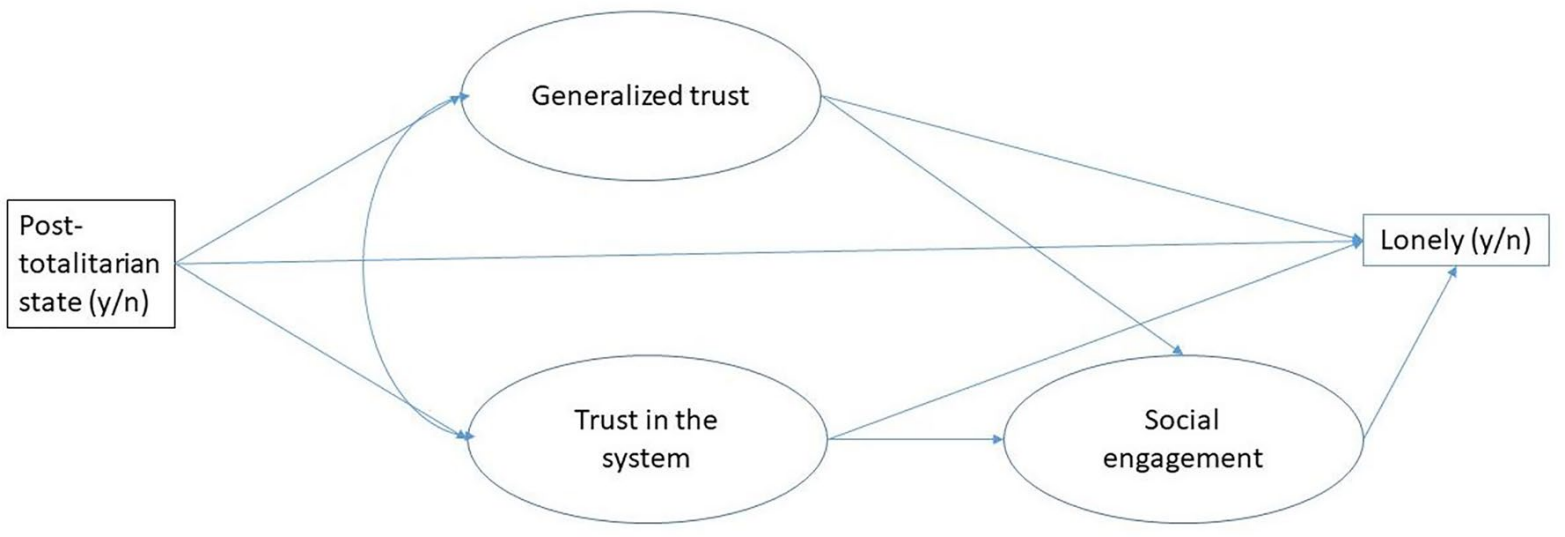
Theoretical model depicting the assumed associations between trust, social engagement, and loneliness

The theoretical model of our study (Fig. 1) is based on a series of assumptions derived from relevant theories. First, we argue that state violence, typical for totalitarian regimes, has shattered trust (Endress and Pabst 2013). Following Luhmann (1968), we distinguish between two interrelated types of trust: personal or generalized trust and systemic trust, or trust in institutions. Based on the social–ecological model discussed in work by Holt-Lunstad (2018), we further expect a direct effect of the type of state on loneliness. Second, trust is considered a key concept in theories about social engagement, either as a proxy of social capital (Coleman 1988; Putnam 2000; Endress 2012, 2014; Carpiano 2006) or as a factor contributing to social engagement (Lin 1999). Trust is crucial for social engagement (Rotenberg et al. 2010; Cottrell et al. 2007; Ernst and Cacioppo 1999), and when social engagement is deficient, loneliness occurs. In sum, our hypothesis is that violence diminishes trust, which increases the likelihood of loneliness directly and through diminished social engagement. Post-totalitarian states are characterized by low trust, and therefore, the prevalence of loneliness is higher in post-totalitarian states compared to non-post-totalitarian states.

Empirical evidence for the different parts of the model comes from various studies. Fenger (2007) observed a strong connection between welfare regime and levels of trust, with the lowest levels of trust in post-communistic states, and the highest levels of trust in social democratic states (i.e. Scandinavian countries). Recent studies observed an inverse relation between levels of trust and feelings of loneliness in older Dutch people (Van Tilburg et al. 2020) and older Finnish people (Nyqvist et al. 2016). There is substantial evidence for an inverse relation between social engagement and loneliness among older Germans (Luhman and Hawkley 2016), older English people (McHugh Power et al. 2019), and older Swedish men (Dahlberg et al. 2015). Empirical evidence for a causal effect of social engagement on trust comes from a study by Jennings and Stoker (2004), who observed based on long-term panel data on three generations of Americans that trust is a cause rather than a consequence of social engagement.

## Data and method

**Fig. 2:**
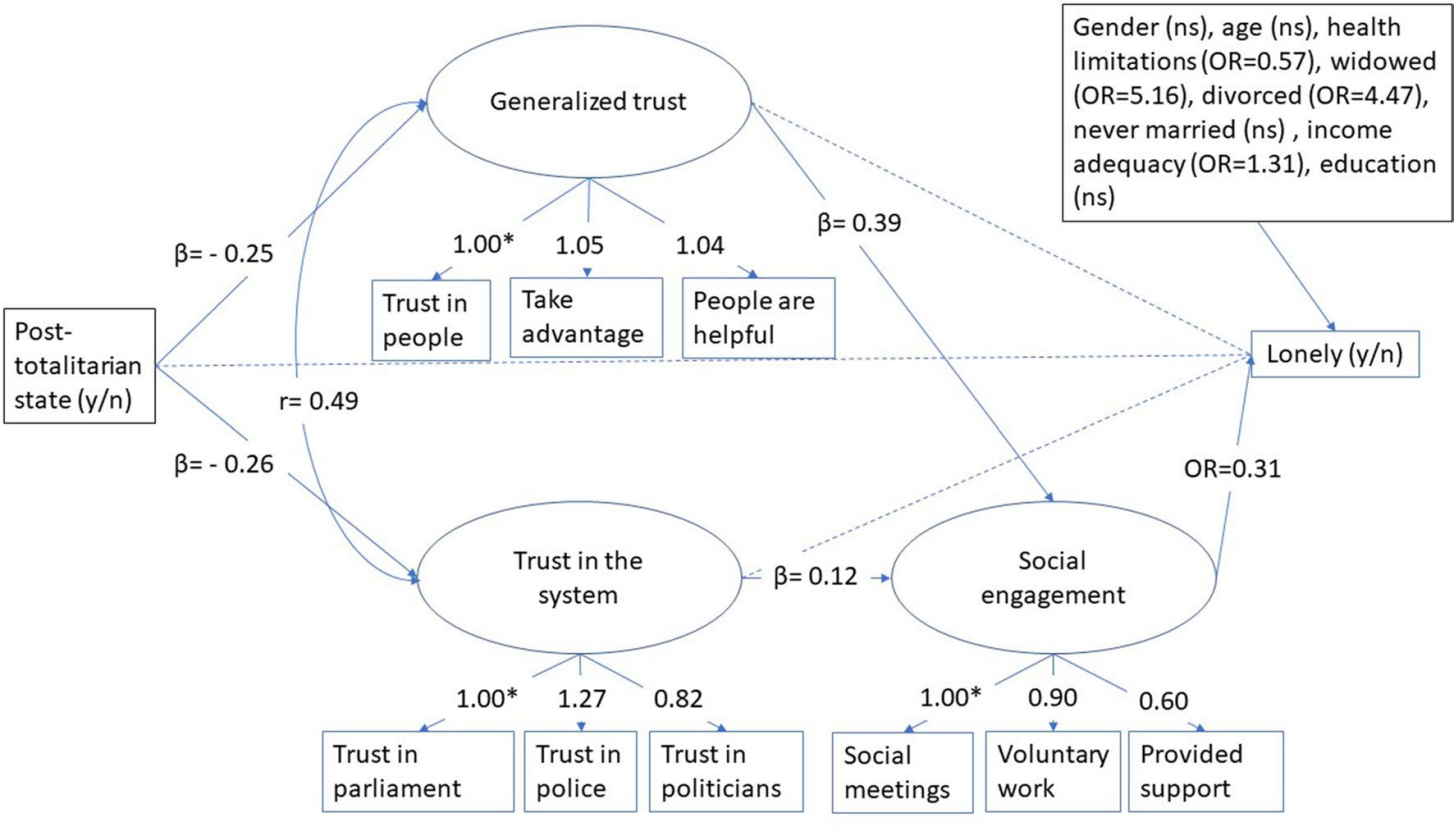
Analytical model and estimated statistics for the associations between the key concepts in our model. A dashed line represents a non-significant association. Note: * Fixed to 1.00, OR = odds ratio, ns = not significant

Data are derived from the European Social Survey (ESS). The ESS is a biennial cross-national survey that has been conducted across Europe since 2001 (https://www.europeansocialsurvey.org/about/). One of the aims of the study is to chart stability and change in social structure, conditions, and attitudes in Europe and to interpret how Europe’s social, political, and moral fabric is changing. The study includes questions on attitudes, beliefs, trust, and behaviour in many European countries and Israel. For our study, we selected the most recent round with the highest number of post-totalitarian countries, and information about loneliness, which was round six (2012). Round six had 29 participating countries, of which 12 were post-totalitarian, whereas round seven (2014) had only 21 countries, six of which were post-totalitarian. The total, unweighted sample of people aged 65 or older in round six comprised 12,042 respondents, of which 4827 live in post-totalitarian countries. For descriptive purposes, we used both the unweighted and weighted sample (Table 1). The analytical model (Fig. 2) was evaluated with the weighted dataset only. In line with the recommendations of the European Social Survey (ESS 2014), we used the post-stratification weight in combination with the population size weights. Respondents with incomplete information on the endogenous variables in the path model are included in the analyses as estimates are based on the robust maximum likelihood (MLR) estimator that is part of Mplus, which allows missingness without leading to biased estimations of the parameters (Muthén and Asparouhov 2002). Respondents with missing information on exogenous variables in the path analyses are excluded by default. This applies to 1.2% (*N* = 188) of the study sample. All variables included in the analytical model are depicted in Fig. 2.

**Table 1:** Loneliness prevalence in 2012 by European countries plus Israel, and post-totalitarianism

	Unweighted sample		Weighted sample*	
Countries	*N*	Prevalence (%)	*N*	Prevalence (%)
*Post-totalitarian European countries*				
Albania	192	28.12	31	19.45
Bulgaria	711	26.02	144	19.47
Czechia	389	23.65	156	21.96
Estonia	602	16.28	24	15.25
Hungary	390	24.1	165	23.93
Lithuania	511	18.98	51	16.56
Poland	342	18.13	571	18.26
Russian Federation	432	31.71	1810	28.55
Slovenia	263	14.45	34	14.37
Slovakia	373	16.62	68	14.43
Ukraine	471	36.09	579	29.24
Kosovo	151	18.54	10	11.59
Subtotal	4827	23.14	3641	25.43
*Other European countries and Israel*				
Belgium	385	13.77	206	14.39
Switzerland	306	4.25	133	4.58
Cyprus	237	18.14	11	15.03
Germany	665	5.11	1604	6.15
Denmark	388	4.12	101	5.01
Spain	371	12.13	738	11.5
Finland	540	7.59	103	7.71
France	534	18.54	1166	14.63
United Kingdom	664	8.73	1067	6.04
Ireland	529	5.29	49	4.14
Israel	450	12.89	82	13.85
Iceland	120	2.5	4	2.63
Italy	187	14.97	1202	16.39
Netherlands	479	7.52	255	5.8
Norway	277	2.17	67	1.87
Portugal	646	18.11	202	13.27
Sweden	437	6.41	176	6.24
Subtotal	7215	9.79	7168	10.23
Total	12,042	15.14	10,809	15.35

*In line with recommendations of the European Social Survey (ESS 2014), we used the post-stratification weight in combination with the population size weights (pspwght*pweight). Source: European Social Survey, Round 6 (2012)

### Dependent variable

Loneliness is measured with a single question: *Please tell me how much of the time during the past week… you felt lonely?* Answering categories are none or almost none of the time (1), some of the time (2), most of the time (3), all or almost all of the time (4). As we are interested in the prevalence of loneliness, we recoded the answering categories 1 and 2 into 0 (not lonely) and categories 3 and 4 into 1 (lonely).

### Independent, intermediate variables and controls

The participating countries were coded 1 (post-totalitarian countries) or 0 (other countries). The *post-totalitarian countries* were Albania, Bulgaria, Czech Republic, Estonia, Hungary, Lithuania, Poland, Russian Federation, Slovenia, Slovakia, Ukraine, and Kosovo. The other countries were Belgium, Switzerland, Cyprus, Germany, Denmark, Spain, Finland, France, UK, Ireland, Israel, Iceland, Italy, Netherlands, Norway, Portugal, and Sweden. *Generalized trust* in people is operationalized as a latent variable based on the following three questions: *Generally speaking, would you say that most people can be trusted, or that you can’t be too careful in dealing with people? Do you think that most people would try to take advantage of you if they got the chance, or would they try to be fair?* And *Would you say that most of the time people try to be helpful or that they are mostly looking out for themselves?* Answering categories range from 1 (indicative of no or low trust) to 10 (indicative of high trust). The latent variable *trust in the system* is based on answers on the following three questions: *Please tell me on a score of 0–10 how much you personally trust each of the following institutions: (1) [country]’s parliament, (2) the police, and (3) politicians.* Answering categories range from 0 (no trust at all) to 10 (complete trust). *Social engagement* is also operationalized as a latent variable and reflects engagement with other people and wider society. Social engagement has three indicators: (1) meeting socially with friends, relatives, or work colleagues, (2) involvement in voluntary work or work for charitable organizations in the past 12 months, and (3) providing help and support to people they feel close to when they need it. Meeting socially is an ordinal variable with seven categories, ranging from 1 (never) to 7 (everyday). Involvement in voluntary work is an ordinal variable with six categories, which we recoded into 1 (never), 2 (once per year), 3 (two times per year), 4 (every three months), 5 (once a month), and 6 (once a week or more often). Support provision was measured with a seven-point Likert scale ranging from 0 (not at all) to 6 (completely).

Control variables were gender, age, health limitations, widowhood, divorced, living alone, income adequacy, and education. *Gender* is a dichotomous variable with male (1) and female (2). *Age* is the number of years alive. *Health limitations* are assessed with a question asking whether people find themselves hampered in daily activities by illness, disability, infirmity, or mental problems, with answering categories “Yes, a lot” (1), “Yes, to some extent” (2), and “No” (3). The legal marital status was used to construct three dummy variables *widowhood, divorced, and never married*. Code 1 was used if the condition applied, the 0 otherwise. To improve the comparison between countries, we use income adequacy rather than absolute income. *Income adequacy* expresses how well people think their income is adequate for their present life, with categories “Living comfortably on present income” (1), “Coping on present income” (2), “Difficult on present income” (3), and “Very difficult on present income” (4). All other answers (refusal, no answer, don’t know) were coded as missing. For *education,* we used the International Standard Classification of Education (ISCED) with seven levels, ranging from “less than lower secondary” (1) to “higher tertiary education” (7). All other values (other, refusal, don’t know, no answer) were coded as missing.

## Statistical approach

We first describe loneliness prevalence among 65+ people in the participating countries for the weighted and unweighted sample. Next, we estimate the analytical model (Fig. 2) to test the hypothesized associations between the key concepts in our theoretical model. The model includes a dichotomous outcome variable loneliness and a dichotomous variable post-totalitarian country. It further includes continuous latent mediators (generalized trust, trust in the system, and social engagement). The control variables gender, age, marital status, health limitations, income adequacy, and education are manifest variables and included in the last step of the estimation of the path model.

A path model with latent variables consists of a measurement part (the estimation of the latent variables) and a structural part (series of linear and logistic regressions to describe the associations between the variables and the total direct and indirect effects). The latent variables are continuous variables that are inferred from the observed variables, similar to factor analysis. In our model, we have three indicators for each latent variable. If the fit of the measurement part was satisfactory according to criteria defined by Hu and Bentler (1999) (i.e. CFI > 0.95; RMSEA < 0.06; SRMR < 0.08), the whole model including the measurement model and the structural model was estimated in the next step. Since we are interested in the loneliness prevalence, we dichotomized the dependent variable loneliness, which implies that the estimated weights for the associations between the variables in the model and loneliness are odds ratios (ORs). For all structural associations, the standardized and unstandardized association was estimated, as well as the 99% confidence interval (CI). A regression weight is significant, if the 99% CI does not include 1 (for ORs) or 0 (for Bs and Betas). The model is estimated with the maximum likelihood estimator with robust standard errors (MLR) using the Monte Carlo integration algorithm with 500 integration points. MLR parameter estimates are robust to non-normality and non-independence of observations (Muthén and Muthén 1998–2015).

The estimation of our model was done with Mplus 8.4 in three subsequent steps. In the first step (Model 1), we estimated the basic model with type of state as sole independent variable and loneliness as dependent variable, to provide an estimation of the overall effect of type of state on the chance to be lonely. In the second step, we added the latent mediators trust in people, trust in the system, and social engagement to evaluate the hypothesized pathways (Model 2). In the final model, we added a number of control variables to check whether the direct and indirect effects of state on loneliness were maintained if we additionally controlled for factors known to be associated with loneliness (Model 3).

## Results

The prevalence of loneliness among the older population is the highest in post-totalitarian European countries (Table 1). The average percentage of lonely people is 23 in post-totalitarian countries, and 10 in other European countries (unweighted data). The percentage of lonely people in post-totalitarian countries is the lowest in Slovenia (14), Estonia (16), and Slovakia (17), and the highest in Ukraine (36) and Russia (32). For the non-post-totalitarian states, we find the lowest percentages of lonely older people in Norway (2) and Iceland (2), and the highest percentages in France (19), Cyprus (18), and Portugal (18).

The fit indices of the measurement model including the three latent variables were all far below the cut-off indicating that the measurement model fitted the data very well (CFI = 0.98; RMSEA = 0.04; SRMR = 0.03). Next, we added the structural paths (in Fig. 2 shown as one-headed arrows) between the variables. We also included a residual correlation (in Fig. 2 shown as curved two-way arrows) between the two latent variables trust in the system and trust in people, to take into account the potential interrelatedness, and to control for the effect of unknown confounding variables (MacCallum et al. 1993).

Table 2 shows the estimates of the three structural models: only country effects (Model 1), full model without control variables (Model 2), and full model with control variables (Model 3). Model 1 indicates that the odds that a person in a post-totalitarian European country is lonely is 2.99 times higher compared to people living in other EU countries (OR = 2.99, *p* < 0.01). Type of state (post-totalitarian versus other types of country in Europe) explains 8% of the variation in loneliness. Model 2 shows that part of the state effect on loneliness is explained by the level of social engagement as the OR becomes smaller (OR = 1.89, *p* < 0.01). There is a direct negative effect of the type of state on trust in the system and trust in other people. People in post-totalitarian countries have lower levels of trust in other people (*B* = − 0.80, *p* < 0.01) and lower levels of trust in the system (*B* = − 1.08, *p* < 0.01) than people from non-post-totalitarian countries. Social engagement is inversely associated with loneliness (OR = 0.15, *p* < 0.01), indicating that the more socially integrated people are, the less likely they feel lonely. In turn, social engagement is partly explained by trust in people and trust in the system, with trust in people having a larger effect (*β* = 0.37, *p* < 0.01) than trust in the system (*β* = 0.18, *p* < 0.01). There is no direct effect of trust in people or trust in the system on loneliness, but there is an inverse indirect effect of trust in people and trust in the system on loneliness through social engagement, indicating that higher levels of trust are associated with higher levels of social engagement and, consequently, with lower levels of loneliness (unstandardized total indirect effect B of general trust on loneliness is − 0.47, *p* < 0.01 and *B* = − 0.23, *p* < 0.01 for trust in the system). Type of country, trust in people, and trust in the system additionally explain 28% of the variation in loneliness. Adding to Model 2, the control variables gender, age, health limitations and partner status, income adequacy, and education (Model 3) resulted in a loss of significance for the direct effect of state on loneliness and the indirect effect of trust in the system on loneliness. In other words, the effect of type of state and trust in the system on loneliness can be attributed to variations in health limitations, partner status, income adequacy, and education between the two types of countries. All other associations remained significant.

**Table 2:** Path analyses of loneliness (yes/no) regressed on EU-region, social engagement, trust in the system and trust in people controlling for gender, age, income, education, health limitations, widowhood, divorce, and never married (*N* = 11,854)

	Model 1				Model 2						Model 3					
	OR	99% CI		*p*	*B*	Beta	OR	99%	CI	*p*	*B*	Beta	OR	99%	CI	*p*
Post-totalitarian state-> Loneliness (1 = yes, 0 = no)*	**2.99**	**2.41**	**3.71**	**0.00**			**1.89**	**1.32**	**2.69**	**0.00**			1.21	0.79	1.86	0.31
*Direct effects*																
State-> Generalized trust					− **0.80**	− **0.23**		− **1.04**	**0.56**	**0.00**	− **0.82**	**0.25**		**− 1.04**	**− 0.61**	**0.00**
State-> Trust in the system					**− 1.80**	**− 0.28**		**− 1.31**	**0.85**	**0.00**	**− 1.04**	**0.26**		**− 1.27**	**− 0.80**	**0.00**
Social Engagement (SE)-> Loneliness							**0.08**	**0.02**	**0.25**	**0.00**			**0.32**	**0.14**	**0.75**	**0.00**
Generalized Trust (GT)-> Loneliness							1.00	0.82	1.23	0.98			0.94	0.79	1.13	0.40
Trust in the system (TS)-> Loneliness							1.08	0.93	1.26	0.21			1.03	0.90	1.17	0.64
Generalized trust (GT)—> Social engagement					**− 0.12**	**− 0.37**		**0.08**	**0.16**	**0.00**	**0.15**	**0.39**		**0.09**	**0.20**	**0.00**
Trust in the system-> Social engagement					**− 0.05**	**− 0.18**		**0.02**	**0.08**	**0.00**	0.04	0.12		0.00	0.08	0.01
*Indirect effects*																
GT-> SE-> Loneliness					**− 0.31**	**− 0.22**		**− 0.47**	**0.14**	**0.00**	**− 0.17**	**0.12**		**− 0.29**	**− 0.04**	**0.00**
TS-> SE-> Loneliness					**− 0.13**	**− 0.10**		**− 0.23**	**0.02**	**0.00**	− 0.17	0.04		− 0.10	0.01	0.05
*Residual correlation*																
Generalized trust with trust in the system (correlation)					**0.47**			**0.42**	**0.53**	**0.00**		0.49		0.43	0.54	0.00
*Control variables*																
Education-> Loneliness													0.93	0.85	1.03	0.02
Income adequacy-> Loneliness													**1.31**	**1.05**	**1.63**	**0.01**
Gender-> Loneliness													1.10	0.76	1.60	0.51
Age-> Loneliness													1.00	0.98	1.03	0.99
Health limitations-> Loneliness													**0.57**	**0.46**	**0.70**	**0.00**
Widowhood-> Loneliness													**5.16**	**3.40**	**7.84**	**0.00**
Never married-> Loneliness													1.60	0.79	3.09	0.18
Divorced-> Loneliness													**4.47**	**2.50**	**8.01**	**0.00**
Explained variance loneliness (*R*2)				0.08						0.36						**0.34**

Significant coefficients at p < .01 are printed in bold

## Discussion

The research question of our study was whether the higher prevalence of loneliness in older age in post-totalitarian Eastern and Central European countries could be (partly) attributed to the lower levels of trust in other people and in the system. We hypothesized that the levels of trust in people and in the system are inversely associated with loneliness, both directly and through altered levels of social engagement. Our results are mainly in line with our expectations. The loneliness prevalence in post-totalitarian countries is with 23% substantially higher than in other European countries, where the average loneliness prevalence is 10%. We observed that generalized trust and trust in the system are significantly lower in the post-totalitarian countries than in other countries. Trust in other people and trust in the system are inversely associated with loneliness through lower levels of social engagement, but only the indirect effect of trust in people remains significant after controlling for age, gender, health limitations, partner status, income adequacy, and education.

We further observed that differences in loneliness prevalence can be attributed to the higher number of widowers in Eastern European countries, which is in line with findings by Fokkema and colleagues (Fokkema et al. 2012), and to socio-economic factors such as income adequacy, education, and health limitations, which is in line with the study by Hansen and Slagsvold (2015). Contrary to the findings of Rotenberg (1994), we found that trust was not directly associated with loneliness. While this may indicate potential differences between younger and older people, it may also be caused by a different operationalization and measurement of trust and loneliness, capturing different aspects. While we used measures of trust in the system and general trust in people, Rotenberg applied interpersonal trust scales for peer relations. Furthermore, Rotenberg used the revised 20-item UCLA loneliness scale (Russell et al. 1980), whereas we used a single question to assess loneliness.

The strength of our study lies in the inclusion of former totalitarian states, which revealed the importance of trust for loneliness research and loneliness interventions. Based on theories about trust and social cohesion or social engagement and insights derived from previous research among children and youth, we innovatively connect loneliness with generalized trust in people and trust in the system. The study contributes to current understanding of the construction of loneliness by showing an association between trust and loneliness in different social–cultural environments. Hence, in addition to well-known individual-level risk factors, loneliness can be considered as being constructed by macro-social factors—such as an environment that is favourable for social engagement—that depends on the historical–political context and lies outside the scope of individual control. Trust in loneliness research introduces a new perspective, different from the cultural individualism versus collectivism perspective as studied by Swader (2018) or Lykes and Kemmelmeier (2014). Trust is associated with politics and history, and hence, the seemingly individual and subjective aspects of loneliness are deeply rooted in a much broader social fabric. Trust also connects loneliness research and political studies, as generalized social trust is associated not only with loneliness, but with confidence in political institutions and satisfaction with democracy (Zmerli and Newton 2008; Uslaner 2002), as well as with economic inequality and corruption (Rothstein and Uslaner 2005; Freitag and Bühlmann 2009).

## Limitations

The quantitative approach and questions about feeling lonely and trust used in the ESS do not give specific information about the conceptions of people and what they actually mean while answering the questions about loneliness (social, emotional, existential) or trust (trust as taking risks, habitual, or other). Questions such as which mode of trust is most important for social engagement, which type of loneliness is reduced by what mechanism, or whether level of trust influences cultural expectations (standards) with respect to loneliness remain unanswered. Moreover, the assessment of loneliness is based on the single question, “Do you feel lonely?”, which may generate socially desirable answers as people do not like to admit that they feel lonely (Victor et al. 2005). However, empirical data show that classifying respondents as lonely when they are often or always lonely as we do in our study is remarkable similar for single questions and aggregated scales (Victor et al. 2005), which limits the potential underestimation of loneliness.

Another limitation is that the ESS is a repeated cross-sectional study. While our reasoning is that the difference in loneliness prevalence is caused by low social engagement and diminished trust typical of post-totalitarian countries, reversed causality between trust, social engagement, and loneliness cannot be ruled out. For example, lonely people may actively distance themselves from social engagement, leading to even stronger distrust of other people (Newall et al. 2009). Moreover, our focus was on differences between countries, which left possible variations within countries undetected. There are most likely also regional or ethnic variations within countries, and levels of trust may be patterned according to region or ethnicity (Abascal and Baldasarri 2015). Cross-national longitudinal studies that take into account potential regional and/or ethnical variations are required to investigate the causal order of the key concepts trust, social engagement and loneliness, and within-country variations in these relations.

Finally, we cannot fully rule out alternative explanations for the associations between post-totalitarian regime and loneliness prevalence. While we control for a number of alternative explanations (e.g. differences in demographics, health, economy, and education), there may still be factors other than trust mediating the relationship between living in a post-totalitarian regime and the risk of loneliness.

## Conclusions

Lack of trust in other people and in the system (parliament, police, and politicians) is an essential precondition for loneliness. While trust cannot be directly linked to loneliness, it is linked through the reduction in social engagement, which is in turn associated with higher risk of loneliness. Since levels of trust vary greatly across European countries, social policies to reduce loneliness should be sensitive to the specifics of particular countries and taken seriously the level of trust people have in the system and in other people. In order to improve the effect of loneliness interventions, a long-term approach is advised, with generalized trust and trust in the system stimulated or developed before loneliness interventions are introduced.

## Data Availability

ESS data are open access.
